# Heparin-free veno-arterial extracorporeal membrane oxygenation in lung transplantation: a retrospective cohort study

**DOI:** 10.1186/s13019-024-02721-y

**Published:** 2024-04-20

**Authors:** ZhaoMin Huang, Jiayi Zheng, Mingyang Wang, Shaoting Zeng, Miaoting Huang, Shuyi Peng, Jiajun Li, Jiaming Ji, Quan Chen, Xin Xu, Chao Yang, GuiLin Peng, Hanyu Yang

**Affiliations:** 1 Department of anesthesia, The First Affiliated Hospital of Guangzhou Medical University, No. 151 Yanjiang Rd, Guangzhou, 510120 China; 2 The First Clinical Medical College, Guangzhou Medical University, No. 195 West Dongfeng Road, Yuexiu District, Guangzhou, 510120 China; 3 Department of Thoracic Surgery, The First Affiliated Hospital of Guangzhou Medical University, No. 151 Yanjiang Rd, Guangzhou, 510120 China

**Keywords:** Veno-arterial extracorporeal membrane oxygenation, Heparin free, Thrombus events

## Abstract

**Background:**

In lung transplantation (LTx) surgery, veno-arterial extracorporeal membrane oxygenation (VA-ECMO) can provide mechanical circulatory support to patients with cardiopulmonary failure. However, the use of heparin in the administration of ECMO can increase blood loss during LTx. This study aimed to evaluate the safety of heparin-free V-A ECMO strategies.

**Methods:**

From September 2019 to April 2022, patients who underwent lung transplantation at the First Affiliated Hospital of Guangzhou Medical University were retrospectively reviewed. A total of 229 patients were included, including 117 patients in the ECMO group and 112 in the non-ECMO group.

**Result:**

There was no significant difference in the incidence of thrombus events and bleeding requiring reoperation between the two groups. The in-hospital survival rate after single lung transplantation (SLTx) was 81.08%in the ECMO group and 85.14% in the Non-ECMO group, (*P* = 0.585). The in-hospital survival rate after double lung transplantation (DLTx) was 80.00% in the ECMO group and 92.11% in the Non-ECMO groups (*P* = 0.095).

**Conclusions:**

In conclusion, the findings of this study suggest that the heparin-free V-A ECMO strategy in lung transplantation is a safe approach that does not increase the incidence of perioperative thrombotic events or bleeding requiring reoperation.

## Introduction

Lung transplantation (LTx) is considered a last resort for patients with severe lung disease, and anesthesia during the procedure poses significant challenges. Extracorporeal life support is frequently employed in lung transplantation (LTx). In comparison to cardiopulmonary bypass (CPB), extracorporeal membrane oxygenation (ECMO)offers the advantages of reduced heparinization, decreased blood [1–3]. Consequently, LTx is now performed either with ECMO or Non-ECMO.

V-A ECMO is an effective treatment for severe pulmonary hypertension, right heart failure, RV dysfunction, and inability to maintain adequate oxygenation of the body depending on lungs during one-lung ventilation. However, using heparin to manage ECMO can increase blood loss during LTx. Bleeding not only increases the difficulty and risk of surgery, but large transfusions of blood products are also a risk factor for severe primary graft dysfunction (PGD) and increase early recipient mortality [4, 5].

During the LTx surgery, our center adopted the heparin-free V-A ECMO management strategy to reduce the adverse effects of systemic heparin. In addition, few successful cases of systemic heparin-free or low-dose heparin LTx have been reported due to the lack of systematic reviews [6]. Currently there is no consensus on anticoagulation strategies for VA-ECMO during LTX surgery.

To evaluate the safety of the heparin-free V-A ECMO strategy, this study retrospectively reviews perioperative data of LTx in comparison with postoperative thrombotic events, reoperation for bleeding, and postoperative early survival of LTx patients in non-ECMO groups and V-A ECMO groups.

## Methods

### Study design and patients

The study was a single-center, retrospective cohort study. From September 2019 to April 2022, a total of 229 consecutive cases of LTx were evaluated, including 117 cases in the ECMO group and 112 cases in the non-ECMO group. The basic information about the patients is shown in Table 1. The data were extracted from the electronic medical records of the First Affiliated Hospital of Guangzhou Medical University. The study was reviewed and approved by the Ethics Committee of the First Affiliated Hospital of Guangzhou Medical University. Informed consent was not required due to the retrospective nature of the study. All donor lungs were provided by Organ Procurement Organizations and were conducted in compliance with Chinese legislation. None of the organs were procured from executed prisoners and that organs were procured after informed consent or authorization.

**Table 1:** Clinical characteristics of lung transplant recipients($\bar{x}$ ± s or n)

Variables	Before Propensity Score Matching			After Propensity Score Matching		
	ECMO group (n = 117)	Non-ECMO group (n = 112)	P	ECMO group (n = 37)	Non-ECMO group (n = 37)	*P*
Gender, males/females	80/37	101/11	0.00	27/10	30/7	0.41
Age, years	55.24 ± 13.27	55.54 ± 11.25	0.04	55.76 ± 12.54	53.08 ± 13.62	0.38
BMI	19.77 ± 4.15	19.62 ± 4.33	0.78	20.01 ± 3.58	19.81 ± 5.24	0.84
Intraoperative blood loss (ml)	1308.12 ± 1588.12	543.03 ± 1003.58	0.00	875.68 ± 950.12	993.76 ± 1626.22	0.70
Intraoperative RBC transfusion(units)	6.96 ± 7.36	1.17 ± 3.07	0.00	4.07 ± 4.95	3.26 ± 4.67	0.47
Operation time(min)	466.98 ± 133.03	318.66 ± 126.74	0.00	375.03 ± 111.84	417.00 ± 139.36	0.16
SLT/BLT(n)	37/80	74/38	0.00	19/18	11/26	0.06
Transplant indication			0.00			0.00
COPD	7(6%)	52(46%)		1(3%)	10(27%)	
Lung fibrosis	14(12%)	6(5%)		5(14%)	0(0%)	
IPF	5(4%)	4(4%)		2(5%)	0(0%)	
Pneumosilicosis	7(6%)	5(4%)		3(8%)	1(3%)	
Bronchiectasis	11(9%)	3(3%)		0(0%)	2(5%)	
NISP	54(46%)	30(27%)		20(54%)	13(35%)	
Others	19(16)	12(11%)		6(16%)	11(30%)	

BMI, body mass index, COPD: chronic obstructive pulmonary disease, IPF: idiopathic pulmonary fibrosis, NSIP: Nonspecific interstitial pneumonia

### Anesthesia and surgical incision

Anesthesia was administered intravenously or intravenously, combined with inhalation. A double-lumen tube (DLT) was used for lung isolation. For SLTx, the DLT was inserted into the non-operative side, while for DLTx, the left DLT was selected to avoid intraoperative DLT replacement. The protective ventilation strategy was used. Intraoperative monitoring included radial artery lines, Swan-Ganz catheter for pulmonary artery pressure, PiCCO (Pulsion Medical System, Munich, Germany) for hemodynamic changes, TEE for cardiac function, vascular anastomosis, and hemodynamic changes. The surgery was performed using a clamshell incision (for DLTx) or an anterolateral chest wall incision (for SLTx).

### Intraoperative ECMO criteria

In the ECMO group, all patients used VA-ECMO except three patients who used VV-ECMO before surgery and switched to VVA-ECMO during surgery.

The intraoperative EMO placement criteria are based on the Hannover team’s guidelines [7]. If, after clamping of the pulmonary artery, any of the subsequent conditions occurred: (1) decrease of arterial saturation less than 90%; (2) cardiac index less than 2l/min/m^2^; and (3) Suprasystemic (pulmonary artery, PA) pressures.

### Perioperative ECMO management

For the femoral artery, a 17Fr arterial infusion tube (MAQUET BE-PAS1715 Art HLS cannula, insertion length 15 cm) was used, and for the femoral vein, a 21Fr venous drainage tube (MAQUET BE-PVL2155 Venous HLS cannula, insertion length55cm) was used. Use heparin-free Ringer’s acetate solution for priming the ECMO circuit. No additional intravenous systemic heparin was administered until the end of the surgery. During surgery, the ECMO flow was set at 2 to 4 L/min, depending on the patient’s hemodynamic status. Try weaning from ECMO at the end of the surgery. If severe reperfusion pulmonary edema or acute PGD develops following transplantation, ECMO support is continued to ICU. A low dose of heparin is administered according to the bleeding condition to maintain the activated clotting time (ACT) within 160 to 180 s or activated partial thromboplastin time (APTT) 50 to 55 s to prevent ECMO-related thrombotic complications.

### Medical record review and survival follow-up

All patients were routinely screened for deep vein thrombosis by Doppler ultrasonography within 24 h after surgery. In this study, thrombotic events within 30 days after LTx surgery were defined as venous and intracavitary arterial thrombosis, pulmonary thromboembolism, myocardial infarction, cerebral thrombosis, and portal vein thrombosis. A manual review of medical records was conducted to confirm all thrombotic events and cases of bleeding requiring reoperation. The patients were followed up on Novenmber.1.2022. Patients who lost to follow-up and for whom survival data were missing were excluded from the study.

### Statistical analyses

Propensity score matching (PSM) was performed to balance the patient characteristics and reduce potential selection bias to the two groups(ECMO group and Non-ECMO group). Using 1: 1 nearest neighbor matching, the caliper value was set to 0.02. The propensity score was calculated for the following variables: age, gender, BMI, intraoperative blood loss, intraoperative RBC transfusion, operation time and type of transplantation(SLT or BLT).

Continuous variables were presented as mean ± standard deviation and compared using the t-test for independent samples. Categorical variables were presented as numbers and were compared using the Chi-square test or Fisher’s exact test. All data were analyzed using SPSS software (SPSS version 25.0; IBM Corp., Armonk, NY, USA). *P* values < 0.05 were considered statistically significant. For survival analysis, Kaplan-Meier survival curves were generated using the statistical software GraphPad Prism (version 8.0).

## Results

The basic characteristics and intraoperative data of the patients are shown in Table 1.

Before PSM, the amount of intraoperative blood loss (1308.12 ± 1588.12 mL vs. 543.03 ± 1003.58mL, *P* < 0.05) and RBC infusion (6.96 ± 7.36 U vs. 1.17 ± 3.07 U, *P* < 0.05) was significantly higher in the ECMO group as compared to the non-ECMO group. the operation time was longer in the ECMO group than in the non-ECMO group (466.98 ± 133.03 min vs. 318.66 ± 126.74 min, *P* < 0.05 ), the incidence of single and double LTx was 37/80 vs. 74/38 (*P* < 0.05). After the PSM, there was no significant difference in general clinical data between the two groups (*P* > 0.05).

After PSM, there was no significant difference in the incidence of thrombus events and bleeding requiring reoperation between the two groups (Table 2).

**Table 2:** Thrombotic events and Bleeding require reoperation

Variables	Before Propensity Score Matching			After Propensity Score Matching		
	ECMO group (n = 117)	Non-ECMO group (n = 112)	*P*	ECMO group (n = 37)	Non-ECMO group (n = 37)	*P*
Thrombotic events(cases)(%)	8(6.84)	4(3.57)	0.27	4(10.81)	1(2.70)	0.36
Bleeding require reoperation(cases)(%)	4(3.42)	0	0.12	1(2.70)	0	1

**Fig. 1:**
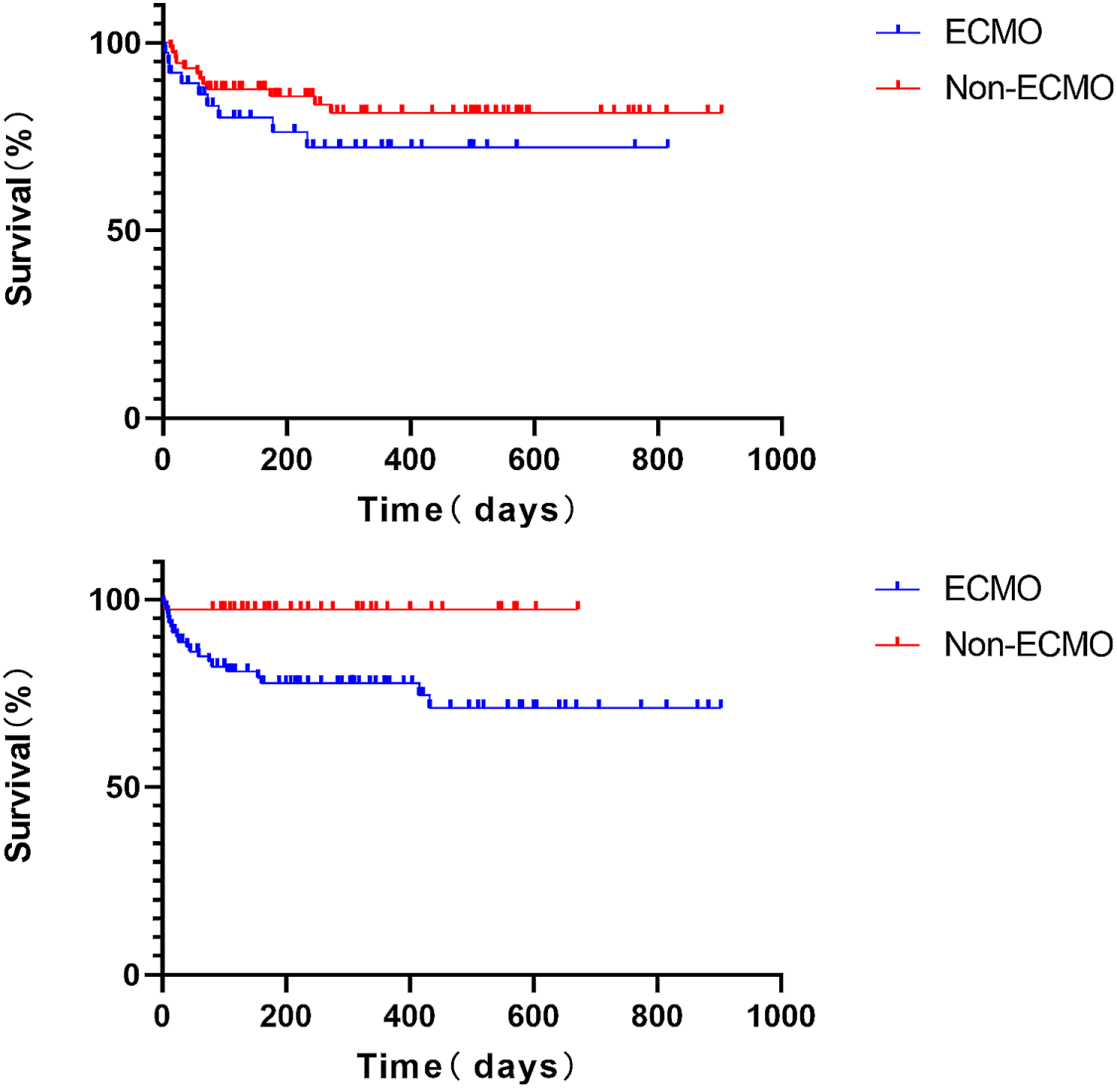
**A**. Single lung transplantation survival curve. **B** Double lung transplantation survival curve

The in-hospital survival rate after single lung transplantation was 81.08% v.s 85.14% in the ECMO and Non-ECMO groups, respectively (*P* = 0.585). The in-hospital survival rate after double lung transplantation was 80.00% vs. 92.11% in the ECMO and Non-ECMO groups, respectively (*P* = 0.095). Kaplan- Meier survival curve analysis (Fig. 1) showed that the SLTx survival in the ECMO group was comparable to that of the non-ECMO (Log-rank test *P* = 0.2636 and Gehan-Breslow-Wilcoxon test *P* = 0.2516). The DLTx survival of the non-ECMO group was better than that of the ECMO group (Log-rank test *P* = 0.0081 and Gehan-Breslow-Wilcoxon test *P* = 0.0123).

## Discussion

ECMO has proven to be a valuable alternative to cardiopulmonary bypass (CPB) in lung transplantation, with favorable survival rates [8, 9]. The use of VA ECMO in lung transplantation has the following advantages: (1) Protective ventilation can be applied to the non-operative lung during lung transplantation, as oxygenation is ensured. (2) It can control and reduce pulmonary reperfusion, reduce pulmonary vascular bed blood flow, and avoid fluid extravasation and further damage to lung function; (3) Stabilize the patient’s hemodynamics [2]. Due to the above factors, in recent years, LTx is usually performed with ECMO or without mechanical assistance.

Bleeding and thrombosis are the most common serious complications of ECMO [10–12], particularly for major procedures such as LTx. In the retrospective study by Hayanga, ECMO as a Bridging Strategy to LTx significantly increased the incidence of reoperation for bleeding by up to 20.41% [13]. In addition, systemic heparinization may increase surgical bleeding and complicate ECMO-supported surgical procedures [14]. The heparin-free ECMO procedure reduces the risk of bleeding. However, few reports of systemic heparin-free or low-dose heparin ECMO lung transplantation and thoracic trauma have been published [6, 15]. In Olson et al.‘s systematic review of anticoagulation-free ECMO, the incidence rates of ECMO circuit thrombosis and patient thrombosis were 13.4% and 19.5%, respectively, while bleeding and major bleeding (severe bleeding) were reported in 32.8% and 27.9%, respectively [11].

The present study is a single-center retrospective study based on real-world data. In this study, the proportion of ECMO in all lung transplant surgeries was 51.10%. To mitigate confounding bias in retrospective studies, we employed PSM. After PSM, there were no statistically significant differences between the two groups in terms of gender, age, BMI, intraoperative blood loss, intraoperative RBC transfusion, operation time and SLT/BLT. The overall incidence of thrombotic events during postoperative hospitalization was 5.24%. Although there was no systematic use of heparin during the operation, the incidence of postoperative thrombotic events was not significantly different between the two groups. The ECMO group had 4 patients (3.42%) who required reoperation due to postoperative bleeding, while no patients in the non-ECMO group needed reoperation for postoperative bleeding. The overall incidence was low in both groups, and there was no statistically significant difference between the two groups before and after PSM. These results confirm that heparin-free ECMO can be used safely and effectively to reduce the risk of postoperative bleeding.

There are no standard protocols for anticoagulation in ECMO support during LTx procedure [14]. Previous studies have shown that short-term systemic heparin-free is safe, reduces bleeding, and does not increase the incidence of thrombosis. In 2009, Hsu et al. [16] first reported 10 successful cases of V-A ECMO support LTx without systemic heparin. In another recent study, Scaravilli et al. reported three successful cases of heparin-free management of V-V ECMO-bridge support during LTx [6]. According to our knowledge, this was the largest retrospective study of heparin-free management of V-A ECMO support during LTx. Our findings are likely attributable to the following reason. First, the heparin-bound ECMO circuits were used in the study, and the ECMO flow was guaranteed to be above 2 L/min during the LTx procedure. Secondly, the intraoperative arterial line, PiCCO catheter, CVP line, and the Swan-Ganz for pulmonary artery pressure measurement were all flushed with a heparin solution (2.5 IU/mL, flow rate 2 ∼ 4mL/h). In addition, heparin saline is needed to prevent clotting in intra-operation samples for blood gases and biochemical tests. In all cases, the operation time was less than 12 h. In conclusion, we believe it is. safe to not use systemic heparin during the operation.

Considering the impact of single and double LTx on the difficulty of the procedure and the difference in survival, this study separated the two groups of patients undergoing single and double LTx in hospitalization and postoperative survival rates. Ius et al. reported that the ECMO group had a higher in-hospital mortality rate than the non-ECMO group. Still, there was no difference in survival rates between the two groups after discharge [7]. This study’s results indicate no difference in in-patient survival between the SLTx or DLTx groups. In addition, the survival curve was comparable between the two groups following SLTx. We believe that the heparin-free ECMO technique may significantly reduce intraoperative bleeding, the incidence of bleeding-related reoperation, and the incidence of postoperative complications, thereby increasing the survival rate. However, the survival curves for DLTx show differences between the two groups. This may be because patients in the non-ECMO group had mild disease and better baseline states before surgery and were able to maintain better hemodynamics and oxygenation during the operation of one lung ventilation without the need for ECMO support during the operation, resulting in a higher postoperative survival rate.

Despite the adoption of the heparin-free technique, the results of this study showed that the ECMO group suffered more intraoperative blood loss and RBC transfusion. This was due to the activation of the coagulation system by surgery and ECMO, coagulation factors, fibrinogen, and platelets. Therefore, coagulation status should be closely monitored during the preoperative period. Patients who require postoperative ECMO support should have their anticoagulation strategy dynamically adjusted according to the chest drainage, coagulation function, and ECMO circuits.

### Limitation

This is a retrospective, single-center study, so systematic bias is difficult to avoid. In this study, the incidence of thrombotic events is not particularly high, which may influence the study results. In addition, due to racial differences, the level of anticoagulation required by Asians may differ from that required by Europeans and Americans. Future multi-center prospective studies with larger samples must provide evidence for clinical work.

## Conclusion

In conclusion, the findings of this study suggest that the heparin-free V-A ECMO strategy in lung transplantation is a safe approach that does not increase the incidence of perioperative thrombotic events or bleeding requiring reoperation.

## Data Availability

The datasets used and analyzed during the current study are available from the corresponding author on reasonable request.
